# Sodium Mercaptoethane Sulfonate Reduces Collagenolytic Degradation and Synergistically Enhances Antimicrobial Durability in an Antibiotic-Loaded Biopolymer Film for Prevention of Surgical-Site Infections

**DOI:** 10.1155/2017/3149536

**Published:** 2017-11-07

**Authors:** Joel Rosenblatt, Ruth A. Reitzel, George M. Viola, Nylev Vargas-Cruz, Jesse Selber, Issam Raad

**Affiliations:** ^1^Department of Infectious Diseases, Infection Control and Employee Health, The University of Texas MD Anderson Cancer Center, Houston, TX 77030, USA; ^2^Department of Plastic Surgery, The University of Texas MD Anderson Cancer Center, Houston, TX 77030, USA

## Abstract

Implant-associated surgical-site infections can have significant clinical consequences. Previously we reported a method for prophylactically disinfecting implant surfaces in surgical pockets, where an antibiotic solution containing minocycline (M) and rifampin (R) was applied as a solid film in a crosslinked biopolymer matrix that partially liquefied in situ to provide extended prophylaxis. Here we studied the effect of adding sodium 2-mercaptoethane sulfonate (MeSNA) on durability of prophylaxis in an* in vitro* model of implant-associated surgical-site infection. Adding MeSNA to the M/R biopolymer, antimicrobial film extended the duration for which biofilm formation by multidrug-resistant* Pseudomonas aeruginosa* (MDR-PA) was prevented on silicone surfaces in the model. M/R films with and without MeSNA were effective in preventing colonization by methicillin-resistant* Staphylococcus aureus*. Independent experiments revealed that MeSNA directly inhibited proteolytic digestion of the biopolymer film and synergistically enhanced antimicrobial potency of M/R against MDR-PA. Incubation of the MeSNA containing films with L929 fibroblasts revealed no impairment of cellular metabolic activity or viability.

## 1. Introduction

All patients proceeding with placement of diverse implantable devices are at risk of infections [1]. Patients with several comorbidities, including cancer [2–4], are usually at higher risk for implant-related infection due to their underlying disease or from other contributory factors including chemoradiation therapy. These implants can be made from metals, diverse polymers, and combinations of these materials. Of interest, patients undergoing breast reconstructive procedures where silicone implants are utilized following mastectomy for breast cancer have an elevated infection rate as high as 23% [5, 6]. Unfortunately, despite numerous preventive measures for these surgeries, including the use of (a) systemic perioperative antimicrobial agents, (b) immersion of the implant or irrigation of the surgical pocket with an antimicrobial solution prior to insertion of the device, and (c) immediate postoperative oral antimicrobials, implant-related complications continue to remain elevated [7, 8]. These include formation of fibrous capsules around the implants as well as complex implant-associated biofilm-related infections that can be difficult to treat with systemic antibiotics alone [9, 10]. Infections of implantable medical devices are particularly problematic [1, 11, 12] because these devices can serve as pathogen reservoirs for recurrent local and disseminated infections eventually requiring implant excision. This can have a devastating effect on patients and increase the economic burden on the health care system [13, 14].

Microbial colonization of implants can cause both inflammation and infection [15, 16]. Colonization-driven inflammation can increase the propensity to form fibrous capsules around implants [17, 18]. Implants can become colonized by microbes during the surgical insertion procedure and postsurgically [15]. Postsurgical colonization can be derived from microbes populating tissue ducts and poor wound healing or through surgical drains that remove fluid accumulating in the surgical pocket following implantation. These complications can be reduced by prolonged localized delivery of appropriate therapeutic agents to the surgical pocket around the implant [19]. Current practices of perioperative irrigation of implants and surgical pockets with aqueous antimicrobial solutions have proven inadequate because the duration where the antimicrobial agents are present is too evanescent [7]. Ideal combinations of therapeutic agents would inhibit microbial colonization without delaying healing.

Previously we reported development of a flexible, meltable, bioabsorbable, antimicrobial film (wrap) that could be placed in the surgical pocket as a solid film but would liquefy in situ to prevent biofilm formation on the implant over durations longer than irrigation with low-viscosity antibiotic solutions [20]. The film delivered minocycline (M) and rifampin (R), an antibiotic combination that has significantly reduced infections associated with vascular catheters and silicone penile implants [21–25]. The approach of delivering therapeutics without incorporating them into the implant shell was used due to the complexity associated with developing and obtaining regulatory clearance for modified implants. Consequently, we sought to develop antimicrobial prophylaxis that did not involve altering implants or their surfaces. The M/R film was capable of protecting silicone from being colonized by Gram-positive and Gram-negative pathogens for 10 days in a simplified* in vitro* model that did not incorporate proteolytic enzymes. Here we used a physiologic* in vitro* model that incorporated collagenase at similar concentrations to those measured in fluids recovered from surgical drains placed following breast reconstruction surgeries. We studied the effect of adding 2-mercaptoethane sulfonate (MeSNA) to the M/R film due to its history of use as a versatile urothelial cytoprotectant in cancer patients [26], as well as its demonstrated ability in* in vivo* studies to potentially reduce fibrous capsule formation [27].

## 2. Methods

### 2.1. Preparation of Solid Films

**Figure 1:**
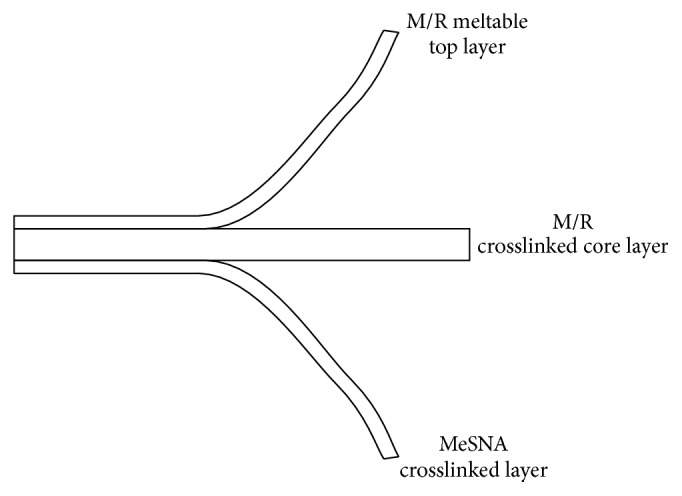
*Diagram of M/R + MeSNA film construction*: M/R bilayer film is first constructed from a crosslinked core layer (0.5 mm thick) with absorbed M/R (from soaking and evaporative drying) and a meltable (noncrosslinked) M/R containing top layer (0.25 mm thick). M/R + MeSNA trilayer films were constructed by pressure laminating a third crosslinked layer (0.25 mm thick) with absorbed MeSNA to the bottom of the core layer of the bilayer film creating the trilayer construction.

Bilayer M/R flexible film laminates consisted of a dehydrothermally crosslinked core (0.5 mm thick) and a noncrosslinked, meltable layer (0.25 mm thick). The layers are comprised of highly plasticized porcine gelatin (plasticized with glycerol). M/R was loaded by swelling in antibiotic solution followed by evaporative drying. Trilayer M/R + MeSNA films consisted of a third MeSNA containing dehydrothermally crosslinked film layer (0.25 mm thick) pressure laminated to M/R bilayer films. Trilaminate M/R films were prepared by laminating a plasticized gelatin film layer (0.25 mm thick) not containing MeSNA to the M/R bilayer film (Figure 1). The final M/R contents in the trilaminates (1 mm thick) were 0.072/0.036%. The final MeSNA concentration in the M/R + MeSNA trilayer laminate (1 mm thick) was 1.8%. The gelatin : glycerol ratio in the M/R and M/R+MeSNA films was approximately 50 : 50. Control gelatin-glycerol films (without antibiotics or MeSNA) were also prepared as controls. All reagents were purchased from Sigma-Aldrich (St. Louis, MO).

### 2.2. Experimental Design

Films were tested in distinct experiments to assess physical changes over time,* in vitro* antimicrobial efficacy and durability, and* in vitro *mammalian cellular cytotoxicity (biocompatibility). Additional studies to elucidate the effect of MeSNA on antimicrobial activity and proteolytic activity in degrading the films were also conducted. Our previous study looked at antimicrobial durability without proteolytic enzyme in the incubating medium [20]. This study modified the method to include collagenase in order to simulate proteolytic degradation of the gelatin-based biopolymer film.

### 2.3. Incubation of Films in Collagenase-Saline Solution at 37°C to Assess Physical Changes

M/R and M/R + MeSNA film samples (1.5 cm × 3 cm segments) were immersed in either 25 ml saline (0.9%) alone of 25 mL of 0.9% saline containing 1 *μ*g/ml collagenase (Sigma-Aldrich, St. Louis, MO) at 37°C. The collagenase concentration, 1 *μ*g/ml, in our* in vitro* model was based on protease levels in patient drain fluid samples following mastectomy [28]. Film samples were weighed periodically over a 2-week duration in order to assess degradation of the films in a simulated postimplantation pocket environment. Saline and saline + 1 *μ*g/ml collagenase were refreshed weekly throughout the duration of testing.

### 2.4. *In Vitro* Antimicrobial Efficacy Testing

Antimicrobial efficacy and durability were examined using an established biofilm colonization model [29]. Briefly, 1 cm diameter silicone discs were cut from medical silicone sheets (Bentec Medical, Woodland, CA) and were covered with either M/R or M/R + MeSNA film. The discs were incubated with 5 × 10^5^ CFU/ml clinical isolates of* methicillin-resistant Staphylococcus aureus* (MRSA) and multidrug-resistant* Pseudomonas aeruginosa* (MDR-PA) in broth containing 1 *μ*g/ml collagenase and incubated overnight at 37°C. These organisms were selected as representative resistant Gram-positive and Gram-negative pathogens causing implant-associated infections in our hospital. Discs were subsequently removed and sonicated in 5 mL of 0.9% sterile saline. After sonication, 100 *μ*L of the resulting liquid was quantitatively cultured and plated onto trypticase soy agar + 5% sheep blood. Plates were incubated over night at 37°C and subsequently counted for growth.

To assess durability, discs were exposed to saline + 1 *μ*g/ml collagenase and tested weekly for up to 2 weeks in the above biofilm colonization model. The saline + 1 *μ*g/ml collagenase was refreshed weekly throughout the duration of testing. Silicone discs sandwiched with control films not containing antimicrobial agents and discs with no film were run as controls. All experiments were performed in triplicate.

### 2.5. Collagenase Enzyme Kinetic Assay

To directly examine the effects of MeSNA on the inhibition of collagenase we used a kinetic assay that examines the breakdown of N-(3-[2-furyl]acryloyl)-Leu-Gly-Pro-Ala (FALGPA) to N-(3-[2-furyl]acryloyl)-Leu + Gly-Pro-Ala in the presence of collagenase. This breakdown can be quantified by continuous spectrophotometric rate determination at *A*_345_ [30]; as FALGPA is degraded to FAL + Gly-Pro-Ala, *A*_345_ decreases. The effect of MeSNA on collagenase was assessed by preexposing collagenase [31] to various concentrations of MeSNA (25 and 10 mg/mL) for 24 hours. FALGPA solution was exposed independently to MeSNA + collagenase or collagenase alone in a 96-well microtiter plates. FALGPA alone with no collagenase was run as a negative control. *A*_345_ was measured every 25 seconds over 5 minutes and plotted over time to determine the rate of FALGPA degradation by collagenase. Results are expressed as the normalized average slope of the linear trendlines fit from the absorbance versus time. All samples were tested with 6 replicates each in 2 experiments.

### 2.6. Assessment of Antimicrobial Synergy

To assess any potential antimicrobial synergy between the M/R and MeSNA components of the films, minimum inhibitory concentration (MIC) and checkerboard assays were conducted. MICs were determined independently for M/R and MeSNA by microbroth dilutions in accordance with CSLI M07 guidelines [32]. MRSA and MDR-PA were exposed to twofold dilutions of M/R (8/4 ug/mL–0.004/0.002 ug/mL) and MeSNA (2,048 ug/mL–0.002 ug/mL). MIC was determined by visual scoring for growth. The well with the lowest concentration of drug in which no turbidity was observed corresponded with the MIC for the organism tested.

Synergy between M/R and MeSNA was assessed using a microbroth dilution checkerboard assay and lowest fractional inhibitory concentration (FIC) analysis as described by the Clinical Microbiology Procedures Handbook [33]. A series of 2-fold dilutions for M/R (8/4 ug/mL–0.125/0.0625 ug/mL) and MeSNA (131,072 ug/mL–128 ug/mL) were combined in a 96-well plates in a checkerboard pattern (one concentration of each drug per row/column) to create unique concentration combinations of the drugs in each well. Test ranges for concentrations of M/R and MeSNA were chosen based on MIC results and concentrations of drugs contained in the films. Synergy was assessed using lowest FIC analysis whereby FIC was determined for all wells along the turbidity/nonturbidity interface and the minimum FIC was used for synergy assessment. An FIC index ≤ 0.5 is considered to be a synergistic combination.

### 2.7. *In Vitro* Mammalian Cytotoxicity

Mouse fibroblast cell line, L929, was selected as it has been used previously in mammalian cytotoxicity testing [34]. Fibroblasts were maintained in Dulbecco's modified Eagle's medium (DMEM) supplemented with 10% heat-inactivated fetal bovine serum (FBS) in 5% CO_2_ at 37°C. Cells were seeded at a density of 4.5 × 10^3^ cells/well in 96-well culture plates for Alamar Blue assay and 2.8 × 10^5^ cells in 25 cm^2^ culture flasks for live/dead staining. When growth reached approximately 60% confluence cells were exposed to a 1%, 0.5%, and 0.25% solution of M/R film extract or M/R + MeSNA film extract in DMEM + 10% FBS for 24 hours. M/R alone or M/R + MeSNA was extracted from films for cytotoxicity testing by placing 2 cm^2 ^M/R or M/R + MeSNA films in 20 mL saline and incubating at 37°C for 48 hrs. DMEM + 10% FBS was used for control, untreated cells. After exposure drug-induced cell viability and toxicity were assessed with Alamar Blue and Trypan staining for live/dead cell exclusion. All experiments were performed in triplicate.

The Alamar Blue assay (Life Technologies, Corp., Carlsbad, CA) was used to assess the sensitivity of fibroblasts to the films. This assay measures the overall metabolic activity of cells based on reduction of resazurin to the highly fluorescent resorufin in response to reductive enzyme activity in cells [35]. Cells sensitive to the experimental drug rapidly lose their ability to metabolically reduce resazurin to resorufin and thus do not produce the fluorescent signal. After 24 hr exposure to M/R or M/R + MeSNA solutions, medium was replaced with 100 uL of Hank's Balanced Salt Solution (HBSS) + 10% Alamar Blue reagent and incubated for 4 hours in 5% CO_2_ at 37°C. Absorbance was determined at 570 nm using a microplate reader spectrophotometer. Cell viability (absorbance) was compared between treated and untreated control cells. Results expressed as a percentage metabolically converted normalized to untreated controls.

The Trypan Blue Exclusion test of cell viability is used to visually determine the number of viable cells present in cell suspension. Live cells with intact membranes have the ability to exclude certain dyes such as Trypan Blue, whereas dead cells do not have the ability. Cells in suspension are stained with 0.4% Trypan Blue and counted on a hemocytometer. Viable cells will have a clear cytoplasm while dead cells will have a blue cytoplasm [36]. After 24 hr exposure to M/R or M/R + MeSNA solutions, cells were washed with HBSS to remove any antitrypsin serum proteins and harvested from the culture flask with 0.05% trypsin EDTA. Once detached, DMEM + 10% FBS was added and cells were pelleted at 200 ×g for 7 minutes. Supernatant was decanted and cells were resuspended in 2 mL of HBSS. Aliquots of 10 uL cell suspension were stained with 10 uL 0.4% Trypan Blue and live and dead cells were counted with a hemocytometer. Results are expressed as percent viable cells in suspension.

### 2.8. Statistical Analyses

Statistical analyses were conducted for comparisons of solutions using Student's *t*-test, two-tailed, unequal variances. Alpha level was set at 0.05 indicating that a *p* value < 0.05 is significant.

## 3. Results

### 3.1. Incubation of Films in Collagenase-Saline Solution at 37°C to Assess Physical Changes

**Figure 2:**
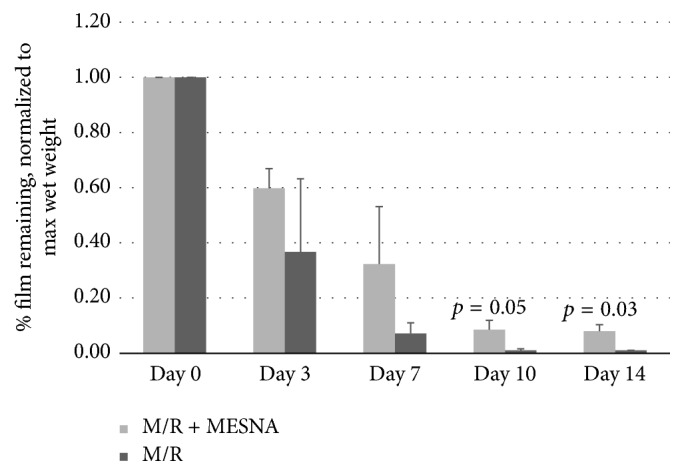
*Durability of M/R and M/R + MeSNA film exposed to 1 ug/mL collagenase*: M/R and M/R + MeSNA films (1.5 × 3 cm) were incubated at 37°C in either saline or saline + 1 ug/mL collagenase. Weights were recorded periodically to assess whether MeSNA inhibited collagenolytic activity.

The measured mass (normalized to maximum mass following immersion in solution) of the M/R and M/R + MeSNA films following incubation in saline or saline + collagenase is presented in Figure 2. Weighing was performed every hour until the films had maximally swelled and then daily for 2 weeks. Fresh collagenase and/or saline was added following weighing at 1 week. The M/R film exposed to collagenase showed a greater loss in weight after 1 week compared to M/R MeSNA film that was not statistically significant (93% versus 68%, resp.; *p* = 0.17). After 10 days, M/R degraded completely while M/R + MeSNA retained 9% of its original wet weight (*p* = 0.05). This persisted to 14 days, where M/R + MeSNA retained 8% of the original wet weight (*p* = 0.03).

### 3.2. *In Vitro* Antimicrobial Efficacy Testing

**Figure 3:**
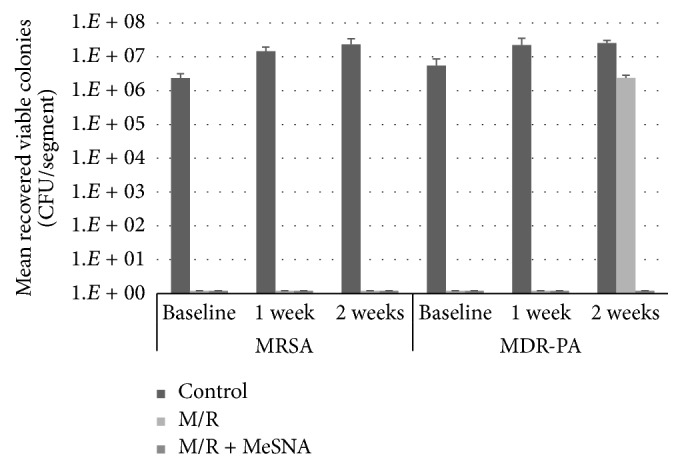
*Effects of MeSNA on antimicrobial efficacy and durability of M/R films*: M/R and M/R + MeSNA films were incubated with 1 ug/mL collagenase and repeatedly tested for antimicrobial efficacy in an antimicrobial durability experiment. Films with M/R + MeSNA had significantly prolonged efficacy (*p* = 0.01) for up to 2 weeks compared to films with M/R alone when tested against MDR-PA.

There were no differences between the control disc + nonantimicrobial gelatin film and control disc without film so only the control disc results are presented as the control in Figure 3. Films containing M/R completely inhibited all challenge organisms from attaching to the silicone at 1 week (*p* = 0.03 for MRSA, *p* = 0.05 for MDR-PA), but breakthrough occurred at week 2 for MDR-PA (at a level of 2.4 × 10^6^ CFU/mL). M/R + MeSNA films completely inhibited attachment of both challenge organisms for 2 weeks (*p* = 0.03 for MRSA, *p* = 0.01 for MDR-PA). Of interest, incorporation of MeSNA visibly decreased the rate of enzymatically driven dissolution of the films.

### 3.3. Collagenase Enzyme Kinetic Assay

**Figure 4:**
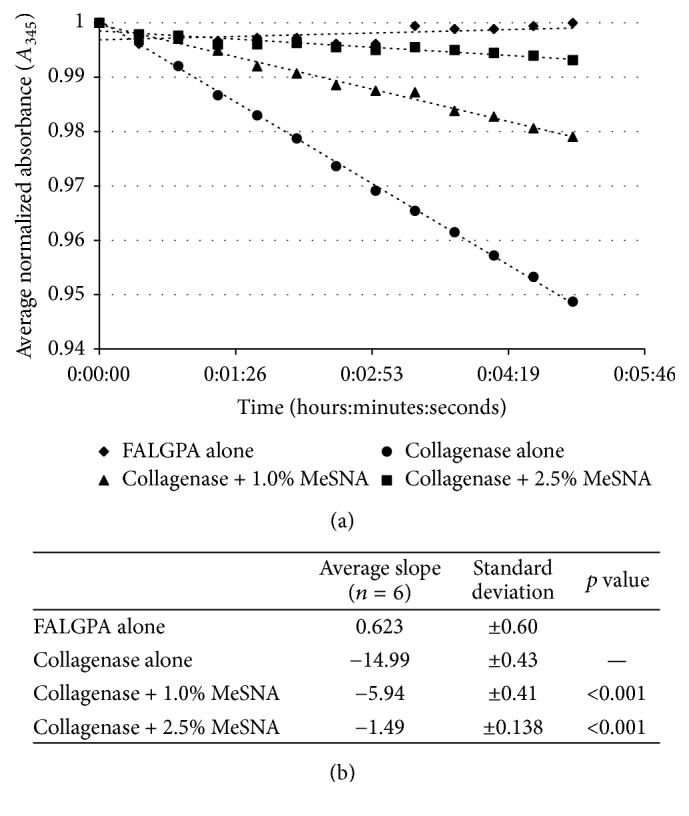
*Degradation of FALGPA by collagenase*: FALGPA was exposed to collagenase and collagenase + various concentrations of MeSNA in a kinetic assay. Absorbance (*A*_345_) readings were measured every 25 seconds for 5 minutes, normalized to time 0, and plotted over time (a) to determine the rate (in absorbance units/min) of FALGPA degradation (b).

The rate of the degradation of FALGPA when exposed to collagenase alone was significantly greater than FALGPA exposed to collagenase + 2.5% MeSNA (rates of −14.99 and −1.49 absorbance units/min, resp. (*p* < 0.001) (Figure 4)). Additionally, as the concentration of MeSNA decreased to 1.0%, the rate degradation increased indicating a dose-dependent response in the ability of MeSNA to inhibit collagenase activity. These results indicate MeSNA directly inhibited proteolytic activity of collagenase.

### 3.4. Assessment of Antimicrobial Synergy

Inhibition of bacterial growth with the M/R combination was tested at a constant 2 : 1 ratio (corresponding to the same ratio of M/R contained in the films). The MIC for MRSA was below the lowest concentrations tested (<0.004/0.002 ug/mL for M/R). Since M/R alone was a highly potent inhibitor of MRSA, synergy with MeSNA was not assessed. For MDR-PA, the M/R MIC was 8/4 *μ*g/mL. The MIC of MeSNA alone was 65,536 *μ*g/mL indicating MeSNA alone had no significant inhibitory activity towards MDR-PA. Fractional inhibitory concentration (FIC) analyses show that the triple combination of M/R + MeSNA was synergistic against MDR-PA (FIC = 0.5) with inhibition at concentrations 4/2 *μ*g/mL M/R and 128 *μ*g/mL MeSNA.

### 3.5. *In Vitro* Mammalian Cytotoxicity

**Figure 5:**
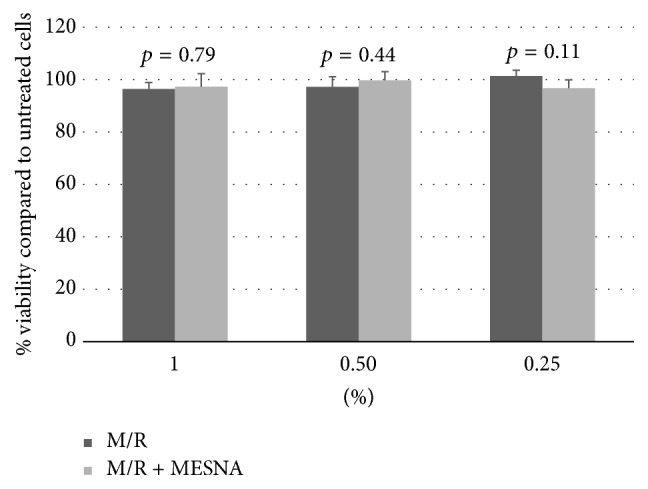
*In vitro cytotoxicity assay*. L-929 fibroblasts were treated with M/R or M/R + MeSNA extracts for 24 hours. Cell viability was assessed with the Alamar Blue assay. Results are expressed as percentage viable cells relative to control untreated cells. No difference in cell viability was detected between M/R and M/R + MeSNA film extracts (*p* > 0.1 for all).

No cytotoxic effects of M/R or M/R + MeSNA extracts were detected with either the Alamar Blue assay or the Trypan Blue Exclusion tests.  Figure 5 shows no significant difference between the metabolic activity of cells exposed to M/R or M/R + MeSNA extracts compared to cells grown in DMEM + 10% FBS (*p* > 0.25 for all M/R extracts and *p* > 0.33 for all M/R + MeSNA extracts tested) or between M/R and M/R + MeSNA films (*p* > 0.1 for all extracts). Additionally, no significant difference was detected for the percent viability in fibroblasts exposed to M/R extract (97.43% viable M/R versus 96.8% viable control; *p* = 0.72) and M/R + MeSNA extract (96.93% viable M/R + MeSNA versus 96.8% viable control; *p* = 0.89) compared to untreated control cells (Table 1).

## 4. Discussion

Surgically implanted gelatin sponges were first utilized for hemostatic control over 50 years ago [37]. Due to proteolytic enzymes, these chemically crosslinked gelatin sponges liquefy* in vivo* within a week or less and are completely absorbed in four-to-six weeks [38]. To simulate this effect in our* in vitro* model we included collagenase at physiologic concentrations, found during postsurgical implant based surgical reconstruction. Furthermore, as surgical drains are typically removed within 10–14 days of breast reconstruction, in order to evaluate the duration of antibiotic protection in a proteolytic environment, we evaluated the effectiveness of antimicrobial M/R + MeSNA films during this high risk period.

MeSNA has been used as a cytoprotective agent with ifosfamide and other chemotherapeutic agents to prevent cystitis. MeSNA, which contains a sulfhydryl group (similar to glutathione), exerts its protective effects by binding to the reactive acrolein metabolite of ifosfamide preventing its hemorrhagic effects [39]. MeSNA has also been used as a mucolytic agent in respiratory therapy where it is thought to exert its mucolytic effects by disrupting disulfide bridges between molecular components of mucus, thereby reducing mucus viscosity [39]. MeSNA is recommended for intravenous administration at concentrations of 2% with daily doses equal to 60% of the daily dose of ifosfamide (up to 1.5 g/m^2^/day) [40]. This concentration is comparable to the final MeSNA concentration in the film but the total dose in the film would be well below what is systemically administered. For short infusion ifosfamide administration, MeSNA is recommended to be administered as three intravenous bolus doses and, for continuous ifosfamide administration, MeSNA is recommended to be administered as one bolus dose and the remainder by constant infusion. MeSNA is too hydrophilic to cross lipid bilayers and enter cells so it is believed to retain presence in extracellular fluid spaces [39]. Therefore, due to this property, we hypothesized that MeSNA would be retained in the surgical pocket for prolonged durations.

M/R was highly potent against MRSA; hence the presence of MeSNA was not needed to make a significant contribution towards enhancing antimicrobial protection against colonization over the two-week course of testing. In contrast, for MDR-PA, where the antibiotic potency was lower, the presence of MeSNA did produce a significant enhancement in antimicrobial protection. Two likely contributory mechanisms towards this result were (a) that MeSNA synergistically enhanced the antimicrobial potency of the M/R combination against MDR-PA and (b) that MeSNA inhibited digestion of the film and therefore the antibiotics were retained at higher concentrations in the vicinity of the silicone. It is likely that both mechanisms worked additively to enhance antimicrobial durability of the MeSNA containing M/R film.

Collagenase and other proteolytic enzymes found in wound settings are metalloproteases. Collagenase has a zinc catalytic center [41–43]. MeSNA is a small molecule with a thiol (SH) group capable of binding to series IIB metal ions such as Zn^+2^ and other metal ions with high affinity [44, 45]. The enzyme kinetic assay showed a significant concentration-dependent inhibition of collagenase activity, likely due to competitive binding at the collagenase catalytic center. Reduced collagenase activity was responsible for much greater mass retention of the film over 2 weeks. We hypothesize that the synergistic enhancement of antimicrobial activity of the MeSNA + M/R combination was a result of the chelating activity of MeSNA.* Pseudomonas species* (and other bacteria) rely on transition metals for key enzymatic and metabolic functions [46, 47]. The combination of chelators and minocycline has previously been shown to have synergistic antimicrobial activity [48], likely a parallel result of the chelator depriving the bacteria of critical metal ions needed for essential growth and survival processes.

Our cytotoxicity studies revealed that the gelatin-based biopolymer film was well tolerated by fibroblasts. This is similar to the biocompatible responses of fibroblasts towards collagen scaffolds [49]. Animal studies have revealed that gelatin sponges assist in the healing process following surgeries [50] without any marked inflammatory or foreign-body response. Gelatin has also been used in conjunction with silicone implants and has been shown to reduce fibrous capsule formation around them [51, 52]. The antibiotics and MeSNA did not impair the favorable biocompatibility of the gelatin-based biopolymer film material.

MeSNA has demonstrated a reduction in capsule formation around silicone implants in rabbit studies. In that study, MeSNA was [27] instilled in the surgical pocket at the time of implantation and capsule thickness measured after 5-month implantation. The MeSNA group had roughly a 50% reduction in capsule and myofibroblast layer thickness around the implants compared to controls. In addition to its antimicrobial activity, minocycline has also demonstrated anti-inflammatory activity [53] mediated by multiple activities including enzyme inhibition and suppression of immune cells [54]. Anti-inflammatory synergy of minocycline and a thiol containing molecule has been reported [55]. Therefore, in addition to the antimicrobial properties of the M/R and MeSNA combination, it is precedent to suggest that it might additionally be beneficial in reducing fibrous capsule formation and contracture around implants as well as postsurgical adhesion and scarring and overall esthetic outcomes.

## 5. Conclusions

Addition of MeSNA to M/R antimicrobial gelatin-based biopolymer laminate film enhanced antimicrobial durability in an* in vitro* physiological implant-associated surgical-site infection model by inhibiting proteolytic absorption of the antimicrobial film and synergistically enhancing the potency of the antibiotics against common microbial pathogens responsible for postsurgical device-related infections. Furthermore, exposure to L929 fibroblasts suggests that the MeSNA containing M/R films may be safe to use clinically. Our hope is that this novel technology will ultimately contribute to decreasing infection and complication rates associated with medical implants; however, further studies in animal models are first needed to confirm our* in vitro* findings.

## Figures and Tables

**Table 1:** *Trypan Blue Exclusion Assay*: L-929 fibroblasts were treated with M/R or M/R + MeSNA extracts for 24 hours. Cell viability was assessed staining harvested cells with Trypan Blue and counted live/dead cells on a hemocytometer were similar among all three groups (*p* = 0.89).

	Untreated L929 cells (cells/mL)	L929 Fibroblasts treated with 2% solution of	
		M/R Film (cells/mL)	M/R + MeSNA Film (cells/mL)
Mean live cells ± standard deviation	1.84 × 10^6^ ± 1.75 × 10^5^	1.39 × 10^6^ ± 1.22 × 10^5^	1.55 × 10^6^ ± 4.19 × 10^5^
Mean dead cells ± standard deviation	6.0 × 10^4^ ± 2.12 × 10^4^	3.80 × 10^4^ ± 3.11 × 10^4^	4.80 × 10^4^ ± 5.66 × 10^3^
% viability	96.89%	97.43%	96.93%
